# An overview of sulbactam‐durlobactam approval and implications in advancing therapeutics for hospital‐acquired and ventilator‐associated pneumonia by *acinetobacter baumannii‐calcoaceticus* complex: A narrative review

**DOI:** 10.1002/hsr2.70066

**Published:** 2024-09-09

**Authors:** Ayush Anand, Amogh Verma, Sarabjeet Kaur, Priyangi Kathayat, Rachel M. Manoj, Aakanksha Aakanksha, Justice K. Turzin, Prakasini Satapathy, Mahalaqua N. Khatib, Shilpa Gaidhane, Quazi S. Zahiruddin, Neelima Kukreti, Sarvesh Rustagi, Arihant Surana

**Affiliations:** ^1^ B. P. Koirala Institute of Health Sciences Dharan Nepal; ^2^ MediSurg Research Darbhanga India; ^3^ Global Consortium of Medical Education and Research Pune India; ^4^ Rama Medical College Hospital and Research Centre Hapur India; ^5^ Government Medical College Patiala India; ^6^ Smt. NHL Municipal Medical College Ahmedabad India; ^7^ Nicolae Testemițanu State University of Medicine and Pharmacy Chisinau Moldova; ^8^ Tbilisi State Medical University Tbilisi Georgia; ^9^ Department of Biomedical Science, College of Health and Allied Sciences University of Cape Coast Cape Coast Ghana; ^10^ Center for Global Health Research, Saveetha Institute of Medical and Technical Sciences, Saveetha Medical College and Hospital Saveetha University Chennai India; ^11^ Medical Laboratories Techniques Department AL‐Mustaqbal University Hillah Iraq; ^12^ Division of Evidence Synthesis, Datta Meghe Institute of Higher Education Global Consortium of Public Health and Research Wardha India; ^13^ Datta Meghe Institute of Higher Education and Research Jawaharlal Nehru Medical College Wardha India; ^14^ Division of Evidence Synthesis, Datta Meghe Institute of Higher Education South Asia Infant Feeding Research Network (SAIFRN), Global Consortium of Public Health and Research Wardha India; ^15^ School of Pharmacy Graphic Era Hill University Dehradun India; ^16^ School of Applied and Life Sciences Uttaranchal University Dehradun India; ^17^ Department of Internal Medicine St. Vincent Hospital Worcester Massachusetts USA

**Keywords:** acinetobacter, durlobactam, hospital acquired pneumonia, sulbactam, ventilator associated pneumonia, Xacduro

## Abstract

**Purpose:**

Infections caused by *Acinetobacter baumannii*, particularly those resistant to antibiotics such as carbapenem, have become a global health crisis with a significant mortality rate. Hospital‐acquired pneumonia (HAP) and ventilator‐associated pneumonia (VAP) resulting from the *A. baumannii‐calcoaceticus* (ABC) complex represent a major clinical challenge. This review aimed to understand the approval process, mechanism of action, therapeutic potential, and future implications of sulbactam‐durlobactam therapy (SUL‐DUR).

**Methods:**

PubMed, Web of Science, EMBASE, Clinical trials. gov, ICTRP, and CENTRAL were searched for studies on SUL‐DUR for the treatment of hospital‐acquired pneumonia and ventilator‐associated pneumonia. Also, World Health Organization, U.S. Food and Drug Administration, and Centers for Disease Control and Prevention websites were searched for relevant information.

**Results:**

SUL‐DUR, marketed as Xacduro, is a novel pharmaceutical combination that functions as a narrow‐spectrum parenterally administered antibiotic. Sulbactam acts as a β‐lactamase inhibitor, whereas durlobactam protects against degradation by *A. baumannii* enzymes. A phase 1 trial successfully established the safety and tolerability of SUL‐DUR in patients with normal and mild renal impairment. A phase 2 trial demonstrated the safety and tolerability of SUL‐DUR in a larger population with urinary tract infections. A phase 3 trial showed that SUL‐DUR was non‐inferior to colistin in terms of mortality in *A. baumannii‐related* VAP, HAP, and bacteremia.

**Conclusion:**

The combination of sulbactam and durlobactam is a promising treatment option for HAP and VAP caused by ABC complex.

## 1 INTRODUCTION

Antimicrobial resistance (AMR) is a global public health threat.^1^, ^2^ A global estimate from 2019 showed approximately 5 million deaths were associated with AMR, with a major burden in developing regions such as sub‐Saharan Africa and South Asia.^3^ Of total AMR‐associated infections, lower respiratory tract infections accounted for approximately 1.5 million deaths, with *Acinetobacter baumannii* being one of the significant pathogens contributing to AMR‐associated mortality.^3^ Specifically, carbapenem‐resistant *A. baumannii* infections (CRAB) were associated with approximately 50,000–100,000 deaths globally.^3^ Patients with CRAB have a significantly higher in‐hospital and 30‐day mortality rate.^4^ A meta‐analysis revealed that patients having infections associated with CRAB are approximately twice as likely to die than those with carbapenem‐susceptible *A. baumannii* infections.^5^ Also, CRAB infections can lead to significantly increased duration of intensive care unit stay.^4^ Various risk factors, such as chronic liver disease, chronic renal disease, hypertension, immunosuppressed state, and intubation, may lead to increased mortality.^6^ Recent studies have also demonstrated a significant association between CRAB infection and extensive drug resistance, which can further complicate the management.^7^, ^8^, ^9^, ^10^ The increasing prevalence of CRAB strains globally points towards a major limitation of existing therapeutic strategies. Furthermore, risk factors for the emergence of multi‐drug and extensive‐drug resistant *A. baumannii* infections include previous exposure to carbapenem, tracheostomy, mechanical ventilation, and intensive care unit utilization.^11^, ^12^ The COVID‐19 pandemic further accelerated the rise of AMR, with CRAB being a significant contributor.^13^, ^14^

Due to the high mortality and AMR associated with CRAB, the Centers for Disease Control and Prevention (CDC) has categorized it as an urgent public threat, and the World Health Organization (WHO) has listed it in the priority pathogen list.^9^, ^15^, ^16^, ^17^ It presents a grave risk to patients in healthcare settings, particularly those reliant on ventilators, as highly resistant *A. baumannii* strains, including carbapenem‐, multi‐, and extensive‐resistant strains, continue to spread. The multi‐resistant and extensive drug‐resistant strains lead to excess morbidity and mortality.^1^, ^3^, ^11^, ^15^, ^16^, ^18^, ^19^, ^20^ In addition to this, the increasing economic burden due to CRAB can be highly challenging, particularly for resource‐poor countries.^12^, ^21^, ^22^

The current treatment options for CRAB primarily include the use of high‐dose ampicillin‐sulbactam in combination with at least one other agent.^23^ Often colistin, tigecycline, and minocycline are used in the treatment of CRAB as they are susceptible in the majority of cases.^24^ A meta‐analysis of randomized controlled trials done on 377 patients revealed that almost 36.2% (95% CI = 23.3%–51.3%) of patients receiving colistin developed nephrotoxicity, and patients on colistin therapy are 2.4 times more likely to develop nephrotoxicity than comparators.^25^ Minocycline and tigecycline are associated with an increased risk of hepatotoxicity.^26^, ^27^, ^28^ Newer drugs like cefiderocol have a limited role in HAP/VAP due to CRAB. Also, limited tissue penetration of colistin, moderate quality evidence on efficacy, and the emergence of colistin and pan‐drug‐resistant strains add to the complexity of management modality.^29^, ^30^, ^31^, ^32^ Overall, an undefined therapeutic dosing, high toxicity, and emergence of resistance are major limitations of existing therapeutic modalities and necessitated innovative approaches.^33^, ^34^, ^35^ One such breakthrough is the SUL‐DUR, marketed as Xacduro, for managing HAP/VAP caused by CRAB. This review focuses on the mechanism of action, safety, efficacy, and future prospects of SUL‐DUR.

## 2 METHODOLOGY

We searched PubMed, Web of Science, EMBASE, Clinical trials. gov, ICTRP, and CENTRAL for studies on SUL‐DUR for the treatment of hospital‐acquired pneumonia and ventilator‐associated pneumonia. Keywords used were “sulbactam,” “durlobactam,” “acinetobacter,” “pneumonia,” “hospital acquired pneumonia,” “ventilator associated pneumonia,” “Xacduro,” “carbapenem resistance,” “antibiotic resistance,” “clinical trial,” “randomized.” These keywords were either used alone or in combination with other keywords to obtain relevant results. Narrative reviews, systematic reviews, meta‐analyses, and original studies relevant to the study objectives were included. Also, WHO, U.S. Food and Drug Administration (FDA), and CDC websites were searched for relevant information. Information was extracted from individual articles as relevant and was represented in the form of text, illustrations, and tables. All of this was under the supervision of the AA and AS.

## 3 MECHANISM OF ACTION OF SULBACTAM‐DURLOBACTAM

**Figure 1:**
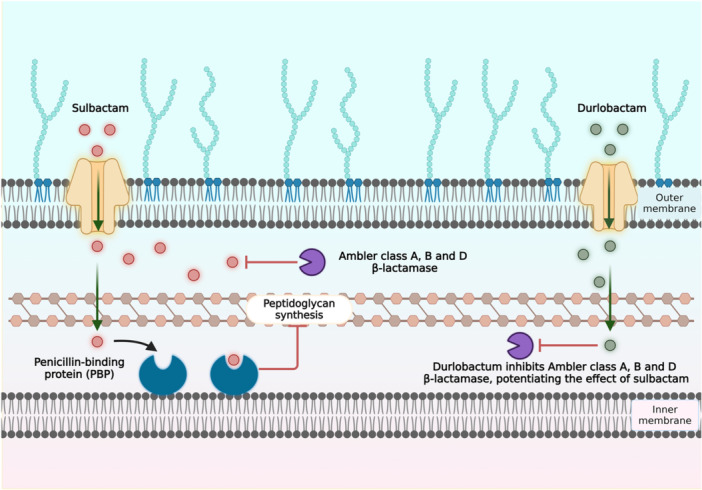
Mechanism of action of sulbactam and durlobactam. [Created with Biorender.com].

On May 23, 2023, the FDA approved Xacduro, a novel SUL‐DUR pharmaceutical combination developed by Entasis Therapeutics.^36^ It is essential to ensure that sulbactam, a penicillin derivative, and durlobactam, a structurally non‐β‐lactam diazabicyclooctane (DBO) β‐lactamase inhibitor, are used in combination. Together, they function as a narrow‐spectrum antibiotic that can be administered parenterally.^37^ Sulbactam acts as a β‐lactamase inhibitor (Figure 1) and exhibits intrinsic activity against *A. baumannii* by inhibiting penicillin‐binding proteins (PBPs) 1 and 3.^37^, ^38^, ^39^ These PBPs play crucial roles as transglycosylases, transpeptidases, and carboxypeptidases in producing peptidoglycan, an essential bacterial cell wall component.^37^, ^40^, ^41^, ^42^ However, sulbactam is susceptible to degradation by various β‐lactamases.^37^, ^43^ Hence, sulbactam has a limited therapeutic role as monotherapy. Durlobactam, on the other hand, is a specifically designed drug that acts as a highly adaptable inhibitor, displaying enhanced reactivity and superior binding to β‐lactamases.^44^, ^45^ This enhancement results from introducing a stronger bond between the 3rd and 4th carbon and adding a methyl group at the third position, distinguishing it from avibactam.^44^ This structural modification enables durlobactam to effectively shield sulbactam against enzymatic degradation, reinstating its efficacy against sulbactam‐resistant ABC isolates expressing these β‐lactamases.^45^, ^46^ The DBO framework of durlobactam has been further altered, enhancing its capacity to inhibit a broad spectrum of class D β‐lactamases and potently inhibiting class A and C β‐lactamases.^39^ Furthermore, a systematic review reported that the SUL‐DUR resistance was seen in only 2.3% of *A. baumannii* inections,^47^ which is far less than cefoperazone/sulbactam (46.3%).^48^ This shows the potential of SUL‐DUR in HAP/VAP.

## 4 SAFETY AND TOLERABILITY OF SULBACTAM‐DURLOBACTAM

SUL‐DUR is primarily cleared renally, which makes it essential to evaluate its safety and tolerability in the setting of renal impairment. To understand this further, a phase 1, open‐label, nonrandomized study involving 34 participants was conducted at three clinical sites in the United States of America between September 2017 and September 2018.^49^ This study assessed the safety of SUL‐DUR in participants with normal, mild, moderate, and severe renal impairment (RI) and those with end‐stage renal disease (ESRD). Safety assessment was done based on the occurrence of adverse events (AEs), vitals, physical examination, electrocardiogram, and laboratory evaluation.^49^ In this study, patients with normal renal function and mild RI did not report any adverse effects. Dizziness, fall, foot fracture, infusion site extravasation, and nausea were reported in participants with moderate RI.^49^ Participants with severe RI reported epistaxis and mucosal dryness.^49^ Also, along with viral upper respiratory tract infection, nausea was reported in subjects with end‐stage renal disease.^49^ Out of 9AEs, six were of mild severity, three were moderately severe, and none of the AEs led to patient discontinuation. However, this trial pointed out the need to adjust dosing in patients with moderate to severe RI and those with ESRD.

To establish evidence for tolerability of SUL‐DUR, a multicenter phase 2 study was conducted from January to May 2018 among 80 hospitalized adults with complicated urinary tract infections, including pyelonephritis.^50^ In this study, SUL‐DUR was given intravenously in the background of imipenem‐cilastatin therapy for 7 to 14 days. A higher rate of AEs (37.7% vs. 29.6%) was observed in the intervention group than in the comparator group. Also, AEs attributable to drug use were higher (22.6% vs. 14.8%) in the intervention group than in the comparator group. AEs included headache, phlebitis, nausea, vomiting, abdominal pain, diarrhea, dyspepsia, gastritis, duodenitis, bronchitis, conjunctivitis, infusion site reaction, vulvovaginal candidiasis, increase in blood glucose and creatinine concentration, elevation of blood pressure and alanine aminotransferase.^50^ Though two patients discontinued intervention due to AEs, no serious AEs and deaths were reported.^50^ Despite the demonstrated safety, the trial did not include HAP/VAP patients with ABC complex, limiting the applicability of the study's findings.

To compare the safety profile of SUL‐DUR with existing treatment strategies, a phase 3 trial evaluated the safety profile of SUL‐DUR in 80 and 81 patients on SUL‐DUR and colistin, respectively.^51^ In this study, the occurrence of any treatment‐emergent adverse event (TEAE) was lower (88% vs. 94%) in the intervention group than comparator group. Specifically, drug‐related TEAEs (12% vs. 30%), serious AEs (40% vs. 49%), and TEAEs (11% vs. 16%) leading to discontinuation, allergic/hypersensitivity reactions (34% vs. 44%), and *clostridium difficile* infections (1% vs. 7%) were lower in the intervention group than comparator group. Since SUL‐DUR is cleared really, nephrotoxicity was the primary safety endpoint, which was assessed based on the RIFLE classification for acute kidney injury.^51^, ^52^ The incidence of nephrotoxicity was significantly lower (13% vs. 38%, *p* < 0.001) in the intervention group than in the control group.^51^ Overall, the safety and tolerability of SUL‐DUR was successfully established based on the findings of various clinical trials.^49^, ^50^, ^51^

## 5 EFFICACY OF SULBACTAM‐DURLOBACTAM

A phase 3 study was necessary to compare the effectiveness of SUL‐DUR with existing therapies. Hence, a phase 3 trial of SUL‐DUR was done in two parts‐ the first part included a randomized cohort of 181 patients and the second part included an observational cohort of 26 patients.^51^ This trial compared the efficacy profile of SUL‐DUR with colistin, in the background of imipenem‐cilastatin therapy given for antibiotic coverage for other causative agents. In the randomized cohort, 91 patients received SUL‐DUR as intervention therapy and 86 patients received colistin as comparator therapy.^51^ SUL‐DUR was given as an intravenous infusion over 3 h every 6 h for 7 to 14 days. Of 91 participants receiving SUL‐DUR, 24 did not complete treatment, and finally, 63 were included for primary efficacy analysis at Day 28. For the colistin group, 31 did not complete treatment, and 62 were included for primary efficacy analysis at Day 28. The 28‐day all‐cause mortality was 12 (19.0%) of 63 in the intervention group and 20 (32.3%) in the comparator group.^51^ This proved the non‐inferiority of SUL‐DUR to colistin in HAP/VAP/bacteremia caused by *A. baumanii*. On adjustment for randomization stratification, the treatment difference was −13.8%, consistent across various subgroups.^51^ Also, the clinical improvement at the therapeutic end point (75% vs. 45%; treatment difference = 29%, 11.4 to 47.4), cure rate (62% vs. 40%; treatment difference = 22%, 2.9 to 40.3), and sustained clinical cure (43% vs. 31%; treatment difference = 12%, −6.2 to 30.6) was higher in the intervention group than control group.^51^ In addition, microbiological outcomes such as response at therapeutic end point (86% vs. 61%; treatment difference = 24%, 7.9 to 40.9), at test of cure (68% vs. 42%; treatment difference = 26%, 7.9 to 44.7) and sustained response at late follow up (48% vs. 40%; treatment difference = 7%, −11.7 to 26.3) was higher in the intervention group than control group.^51^ All these findings provide quality evidence on the non‐inferiority of SUL‐DUR to colistin and advocate for successful use in HAP/VAP and bacteremia due to *A. baumanii*. However, the use of imipenem‐cilastatin in all patients as a background therapy may lead to exaggeration of the actual efficacy of SUL‐DUR. This use of background therapy such as imipenem‐cilastatin with SUL‐DUR raises important concerns about whether these drugs should always be used in conjunction. Hence, a more appropriate study design is mandated for the accurate determination of SUL‐DUR efficacy, which may be limited due to practical considerations of benefit and harm to the patient. Though this may be difficult to achieve using randomized study design, prospective or retrospective studies may help address this issue.

## 6 CLINICAL TRIALS AND APPROVAL PROCESS OF SULBACTAM‐DURLOBACTAM

Approval of SUL‐DUR has witnessed certain significant challenges (Table 1). Findings from the phase 1 study showed SUL‐DUR as a safe and tolerable therapeutic option in healthy subjects or those with mild RI.^49^ However, due to adverse events reported in patients with moderate to severe RI and ESRD, dosing needed to be adjusted.^49^ To further understand the safety and explore the efficacy profile, a Phase 2 study was conducted.^50^ Though it was established that the safety and efficacy profile of SUL‐DUR is similar to colistin, there is a slightly increased incidence of adverse events in the SUL‐DUR group as compared to the control group.^50^ A phase 3 ATTACK trial was conducted to generate quality evidence. This trial showed a lower incidence of adverse events in the SUL‐DUR group, putting rest to safety concerns of the phase 2 trial.^50^, ^51^ Also, the clinical and microbiological assessment showed promising evidence for the efficacy of SUL‐DUR and established non‐inferiority of SUL‐DUR to colistin.^51^ Based on the evidence from the clinical trials, the FDA approved SUL‐DUR for use in the treatment of HAP/VAP caused by susceptible isolates of *A. baumannii‐calcoaceticus* complex, for patients aged ≥18 years.^36^ In the process, the FDA granted SUL‐DUR fast track, priority review, and generating antibiotic incentives now (Table 2) designations.^36^

**Table 1:** Clinical trials on Xacduro.

Parameter	O'Donnell et al.^49^	Sagan et al.^50^	Kaye et al.^51^
Study design	Phase 1, open‐label, nonrandomized study	Phase 2, double‐blind, randomized, placebo‐controlled study	Phase 3, multicenter, randomized, non‐inferiority, open label study
Trial identification	Not applicable	NCT03445195	NCT03894046
Study objectives	To determine the tolerability of SUL‐DUR therapy	To evaluate the safety and efficacy of intravenous SUL‐DUR in the treatment of hospitalized adults with complicated urinary tract infections, including acute pyelonephritis	To compare the safety and efficacy of SUL‐DUR versus colistin in adults aged 18 years or older with ABC complex confirmed HAP, VAP, or bloodstream infections
Study type	Standard design	Interventional (Clinical trial)	Interventional (Clinical trial)
Study period	September 2017–May 2018	January 2018 to April 2018	September 2019 to July 2021
Study setting	Three clinical sites in the United States of America	United States of America	59 clinical sites in 16 countries
No. of study participants	34	80	125
Primary outcome(s)	Safety was assessed from reports of AEs, vital signs, physical examination, 12‐lead ECG, and clinical laboratory testing	Tolerability profile using TEAEs and detailed clinical evaluation Proportion of patients with an overall success (clinical cure and micro‐biologic eradication) for m‐MITT population at the test of cure visit	28‐day all‐cause mortality in patients with laboratory‐confirmed carbapenem‐resistant ABC Nephrotoxicity
Key findings	Six of 9 AEs were mild in severity, and 3 AEs were moderate in severity	The overall rates of success in the m‐MITT population were 36 (76.6%) patients in the SUL‐DUR group and 17 (81.0%) patients in the placebo group Overall success in the microbiologically evaluable population occurred in 36 (80.0%) patients in the SUL‐DUR group and 17 (81.0%) patients in the placebo group	28‐day all‐cause mortality was 12 (19%) of 63 in the SUL‐DUR group and 20 (32%) of 62 in the colistin group Nephrotoxicity was significantly lower in SUL‐DUR than colistin (13% vs 38%, *p* < 0.001)
Adverse reactions	Six (17.6%) subjects experienced 9 AEs, namely, 3 (50.0%) subjects with moderate RI, 1 (12.5%) with severe RI, and 2 (33.3%) with ESRD on HD No healthy subjects or subjects with mild RI experienced an AE	The most reported drug‐related adverse events were headache, diarrhea, nausea, and phlebitis Incidence of TRAEs was 12 (22.6%) patients treated with SUL‐DUR and 4 (14.8%) patients treated with placebo One (1.9%) patient in the SUL‐DUR group had severe nausea, which was considered treatment related and did not lead to discontinuation	Serious AEs were reported in 36 (40%) of 91 patients in the SUL‐DUR group and 42 (49%) of 86 patients in the colistin group TRAEs leading to study drug discontinuation were reported in ten (11%) of 91 patients in the SUL‐DUR group and 14 (16%) of 86 patients in the colistin group
Implications	SUL‐DUR combination is safe to use, however, dosing regimen needs adjustment in the setting of severe renal impairment	SUL‐DUR combination is well tolerated in hospitalized adults in combination with antibiotic coverage for other causative agents	SUL‐DUR combination is safe and efficacious in HAP and VAP in combination with antibiotic coverage for other causative agents

Abbreviations: AE, adverse event; ECG, electrocardiogram; ESRD, end stage renal disease; HAP, hospital acquired pneumonia; HD, hemodialysis; m‐MITT, microbiologically modified intent to treat; RI, renal impairment; SUL‐DUR, sulbactam‐durlobactam; TRAE, treatment emergent adverse events; VAP, ventilator associated pneumonia.

**Table 2:** FDA approval designation of Xacduro.

Designation	FDA definition
**Fast Track** a	A process designed to facilitate the development and expedite the review of drugs to treat serious conditions and fill an unmet medical need. The purpose is to get important new drugs to the patient earlier.
**Generating Antibiotic Incentives Now** b	It creates incentives for the development of antibacterial and antifungal drug products that treat serious or life‐threatening infections.
**Priority Review** c	To act on an application within 6 months (compared to 10 months under standard review). It will direct overall attention and resources to the evaluation of applications for drugs that, if approved, would be significant improvements in the safety or effectiveness of the treatment, diagnosis, or prevention of serious conditions when compared to standard applications.

Abbreviation: FDA, Food and Drug Administration.

^a^ Source‐ https://www.fda.gov/patients/fast-track-breakthrough-therapy-accelerated-approval-priority-review/fast-track (accessed Jan 5, 2024).

^b^ Source‐ https://www.fda.gov/regulatory-information/search-fda-guidance-documents/qualified-infectious-disease-product-designation-questions-and-answers (accessed Jan 5, 2024).

^c^ Source‐ https://www.fda.gov/patients/fast-track-breakthrough-therapy-accelerated-approval-priority-review/priority-review (accessed Jan 5, 2024).

## 7 INDICATION, CONTRAINDICATION, WARNING, AND PRECAUTIONS FOR SULBACTAM‐DURLOBACTAM USE

**Figure 2:**
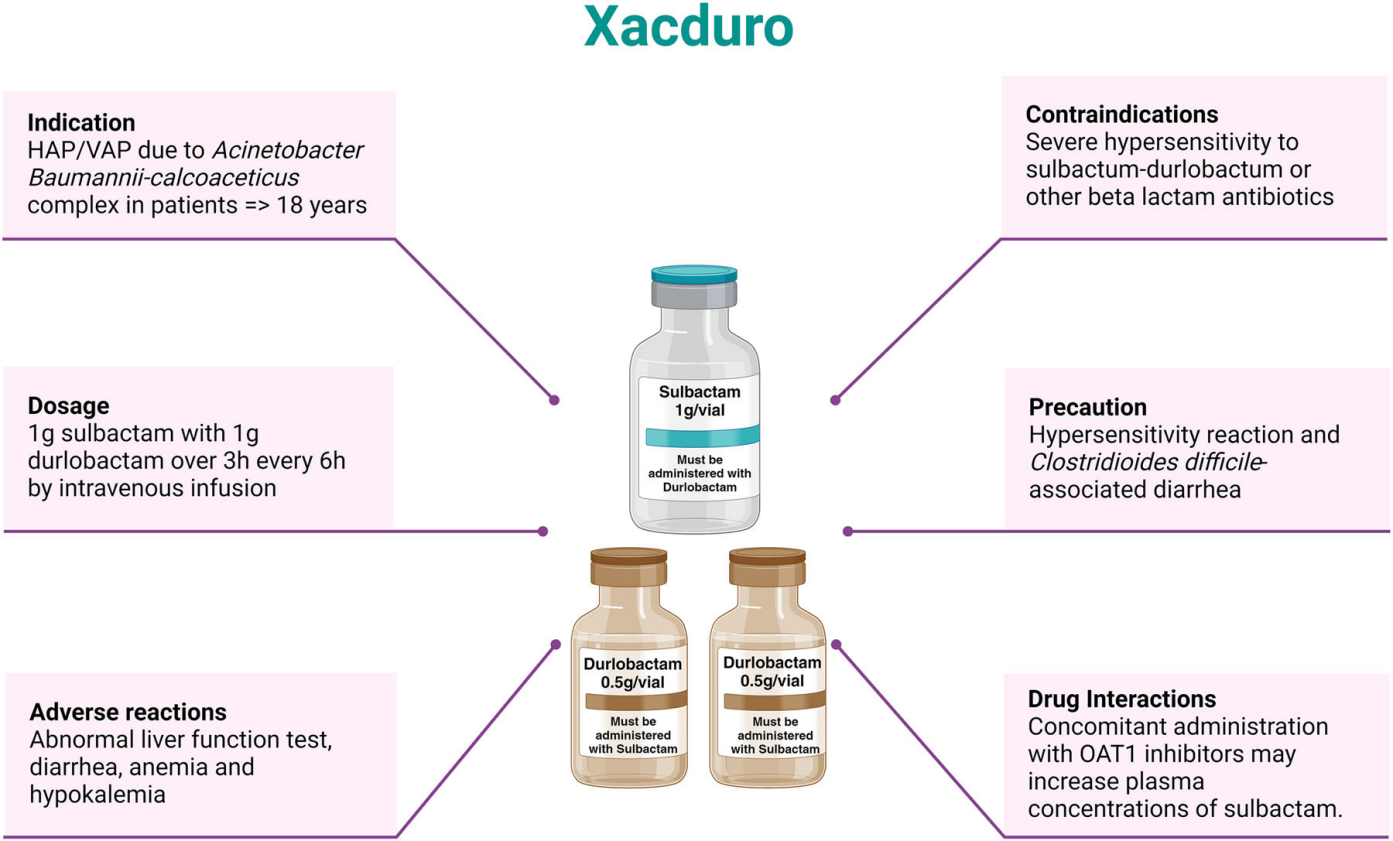
Drug profile of Xacduro. [Created with Biorender.com].

Each carton of SUL‐DUR contains sulbactam in one clear, single‐dose vial, 1 g/vial; and durlobactam in two amber, single‐dose vials, 0.5 g/vial each (Figure 2). Each vial should be reconstituted with sterile water and then injected into an infusion bag for therapeutic use. The standard dosage is 1 g of sulbactam and 1 g of durlobactam given via intravenous infusion over 3 h every 6 h in patients with creatinine clearance (crCL) of 45 to 129 ml/minute.^53^ SUL‐DUR is contraindicated in patients with a known history of severe hypersensitivity to SUL‐DUR or any beta‐lactam antibiotics. Hence, caution should be maintained and detailed drug and allergy history should be taken into account. In addition, it is essential to consider the risk/benefit particularly if clostridiodes difficile‐associated diarrhea is suspected or confirmed, as this can be extremely challenging to manage.^54^, ^55^, ^56^ In addition, judicious use of SUL‐DUR is essential to reduce the chances of AMR. Hence, it should be indicated only in case of an established diagnosis or strong suspicion. Also, clinicians should understand the possible interaction between organic anion transporter 1 (OAT1) inhibitors and SUL‐DUR leading to increased sulbactam concentration, as reported with other beta‐lactam antibiotics.^57^ In addition, clinicians should be vigilant as a significant proportion of patients can have adverse reactions.

## 8 SULBACTAM‐DURLOBACTAM WITH OTHER FIRST‐LINE ANTIBIOTICS FOR ACINETOBACTER INFECTIONS

Though there is no clear consensus on the management of HAP/VAP due to CRAB, the Infectious Disease Society of America has specifically stated recommendations for the management of moderate to severe HAP/VAP.^23^, ^30^ A combination therapy of high‐dose ampicillin‐sulbactam and another agent, irrespective of antimicrobial susceptibility is the recommended approach.^23^, ^58^ Alternatively, polymyxin B/tetracycline/cefiderocol derivatives can be used in conjunction with another agent.^23^ However, cefiderocol is usually reserved for refractory CRAB infections.^23^ High‐dose, extended‐infusion meropenem or imipenem‐cilastatin, rifamycins, and nebulized antibiotics are not recommended.^23^ SUL‐DUR approval comes as a welcome addition to managing cases, especially in the setting of carbapenem resistance. This in combination with antibiotic coverage for other causative organisms, adds a new dimension to the management of HAP/VAP. However, it is very early to establish the combination of SUL‐DUR with other established treatment modalities. Further studies are warranted to compare the safety and efficacy of SUL‐DUR with existing therapeutic modalities. In addition, the cost of procuring antibiotics is a major concern for effective management, particularly in resource‐poor countries. SUL‐DUR is priced at approximately 510 US dollars per carton, taking it to approximately 14,280 US dollars for 1 week of treatment, which is unaffordable for most.^59^ Further studies are required to shed light on cost‐effective management strategies for CRAB infections.

## 9 LIMITATIONS AND RECOMMENDATIONS

Despite the promising potential, It is essential to recognize that the studies on sulbactam and durlobactam for treating HAP and VAP have certain limitations. The phase 2 trial also had certain limitations as it did not add substantial evidence regarding the efficacy of SUL‐DUR. Also, the trial did not include HAP/VAP patients with ABC complex, limiting the applicability of the study's findings. The phase 3 trial only studied 28 days all‐cause mortality and reporting of severe adverse events up to a maximum of 44 days. However, this shorter follow‐up may not be sufficient to account for long‐term renal impairment secondary to intervention. Also, there is a paucity of studies or trials involving individuals with comorbidities such as cardiovascular disease, high blood pressure, diabetes, osteoarthritis, hypercholesterolemia, dyslipidemia, and other conditions. The low representation of women and various racial and ethnic groups does not help in establishing the generalizability of the study's findings. Also, the study did not include pregnant women and cancer patients, which can limit the applicability of the study's findings to vulnerable populations. Another aspect is considering the effect of alcohol on the efficacy of sulbactam. Furthermore, the different genetic strains may respond differently to SUL‐DUR, which needs to be accounted for.

**Figure 3:**
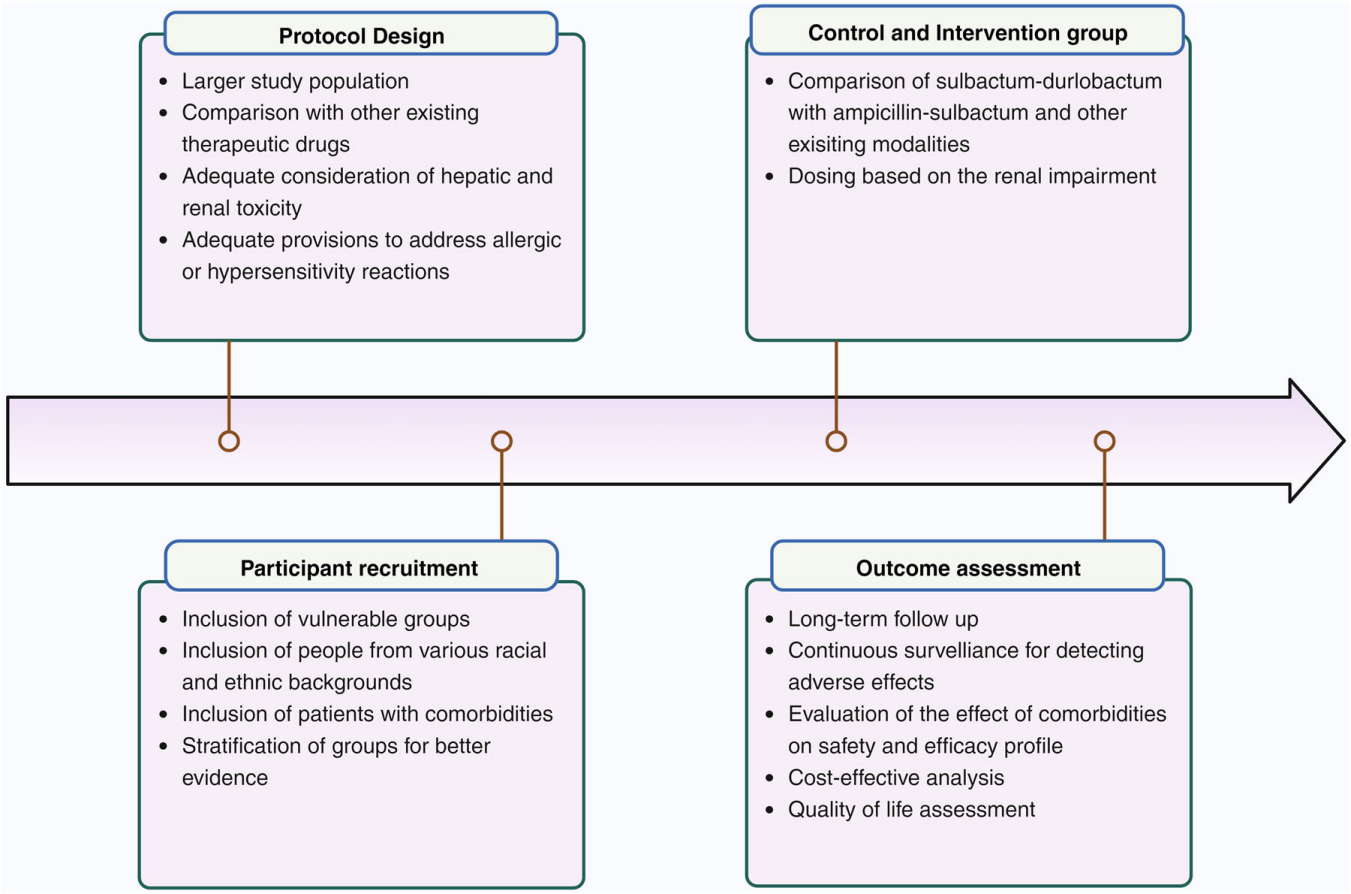
Recommendations for further research. [Created with Biorender.com].

More trials are needed to address allergic or hypersensitivity reactions, as individuals administered sulbactam and durlobactam in the ATTACK trial exhibited a higher frequency of these reactions compared to the control group (Figure 3). Also, additional experimentation and testing are necessary to determine the optimal dosage of medication for individuals with severe renal insufficiency and end‐stage renal disease. A comprehensive study should investigate the effects of sulbactam and durlobactam treatment on individuals who frequently consume alcohol, as evidence suggests that alcohol consumption may interfere with the efficacy of sulbactam. Furthermore, it would be beneficial for the study to include subjects who engage in smoking and alcohol consumption. This would allow for a comprehensive evaluation of the combined impact of these factors on a patient's physical well‐being during treatment.

## 10 CONCLUSION

The increasing prevalence of *Acinetobacter* infections, particularly in healthcare settings and among ventilator‐dependent patients, presents a significant global health concern. Established antibiotic treatments have faced formidable challenges due to the emergence of drug‐resistant *A. baumanii* strains, especially carbapenem‐resistant varieties. The advent of sulbactam‐sulbactam combination therapy represents a promising breakthrough in the fight against CRAB infections, including those resistant to a wide range of drugs, such as multidrug‐resistant and extensively drug‐resistant strains. However, further research and careful consideration of specific patient populations is essential to fully realize this innovative treatment's potential.

## AUTHOR CONTRIBUTIONS

**Ayush Anand**: Conceptualization; methodology; project administration; resources; supervision; validation; visualization; writing—original draft; writing—review and editing. **Amogh Verma**: Writing—original draft; writing—review and editing. **Sarabjeet Kaur**: Writing—original draft; writing—review and editing. **Priyangi Kathayat**: Writing—review and editing; writing—original draft. **Rachel M. Manoj**: Writing—original draft; writing—review and editing. **Aakanksha Aakanksha**: Writing—original draft; writing—review and editing. **Justice K. Turzin**: Writing—original draft; writing—review and editing. **Prakasini Satapathy**: Supervision; validation; writing—review and editing. **Mahalaqua N. Khatib**: Supervision; validation; writing—review and editing. **Shilpa Gaidhane**: Writing—review and editing; validation; supervision. **Quazi S. Zahiruddin**: Supervision; validation; writing—review and editing. **Neelima Kukreti**: Writing—review and editing; validation; supervision. **Sarvesh Rustagi**: Validation; supervision; writing—review and editing. **Arihant Surana**: Supervision; validation; writing—review and editing. All authors have read and approved the final version of the manuscript.

## CONFLICT OF INTEREST STATEMENT

The authors declare no conflict of interest.

## GUARANTOR

Ayush Anand and Arihant Surana are the Guarantors.

## PERMISSION

The figures have been prepared with Biorender, and the publication license for publishing in journals has been obtained, and it is available to be produced at the editor's request.

## TRANSPARENCY STATEMENT

The lead author Ayush Anand affirms that this manuscript is an honest, accurate, and transparent account of the study being reported; that no important aspects of the study have been omitted; and that any discrepancies from the study as planned (and, if relevant, registered) have been explained.

## Data Availability

Ayush Anand had full access to all of the data in this study and takes complete responsibility for the integrity of the data and the accuracy of the data analysis.
